# Supplementary material for: Are carbon emissions trading and green financial instruments synergistic? -Comprehensive quantitative research based on content analysis
Source: PLoS One. 2024 Mar 7;19(3):e0298601. doi: 10.1371/journal.pone.0298601 (PMC10919601; doi:10.1371/journal.pone.0298601)
Supplement: S1 Data — (ZIP) [file pone.0298601.s001.zip › Data/地级市Z_F工作报告.zip!/╣π╬≈/╖└│╟╕█╩╨/└┤╫╘╣1⁄2╓┌║┼í╛┬φ┐╦╩2╛▌═°í┐_2008.docx]

各位代表：

现在，我代表市人民政府向大会作政府工作报告，请予审议，并请各位政协委员和其他列席人员提出意见。

经济社会发展跃上新台阶

2007年，在市委的正确领导下，在市人大、市政协的监督支持下，市人民政府认真贯彻落实党的十七大精神、自治区第九次党代会精神和自治区党委、自治区人民政府的重大决策部署，坚持以科学发展观统领全局，抢抓广西北部湾经济区开放开发等重大机遇，紧紧围绕“三个率先”和市四届人大二次会议确定的目标任务，加快富裕文明和谐防城港建设。全市经济在2006年实现“双跨越”目标的基础上，继续保持又好又快的发展势头，主要经济指标增幅排在广西前列，年初确定的各项目标任务全面完成，经济发展“23124”目标顺利实现。全市社会和谐，边疆安定，民族团结，人民安居乐业。

经济保持高速增长，综合实力进一步增强。地区生产总值突破150亿元，达到159.07亿元，增长20.3%，增速排全区第一，创1995年以来同期最高增幅。财政收入突破15亿元，达到15.76亿元，增长48.8%，增幅排全区第一。产业结构进一步优化，三次产业比例由上年的21.6:42.7:35.7调整为18.8 :48.0 :33.2。城镇居民人均可支配收入12159元，增长33.4%；农民人均纯收入3791元，增长19.5%，是建市以来人民群众生活水平提高最快的一年。社会消费品零售总额30.84亿元，增长18.4%。预计万元生产总值能耗下降1.6%，化学需氧量和二氧化硫排放量控制在自治区要求范围内。

工业发展势头强劲，主导格局基本形成。工业总产值突破200亿元，达到227.57亿元，增长50.3%，其中：规模以上工业总产值183.69亿元，增长61.3%，增幅排全区第一。规模以上工业企业实现利税13.63亿元，增长49.5%；经济效益综合指数达319.64%，居全区首位。工业对经济增长的贡献率达到56.0%。粮油加工、冶金、制糖、化工等重点行业快速发展。全市产值超亿元的工业企业比上年增加14家，达到27家。中电防城港电厂、惠禹饲料蛋白等一批工业项目顺利投产。益海大豆、农垦防城分公司精制糖等项目开工建设。企沙、港口渔洲城和公车、防城河西和茅岭、上思城西、东兴江平等工业园区规划建设加快推进。

港口货物吞吐量突破5000万吨，龙头港口地位进一步增强。防城港20万吨级码头及配套航道和13—15号泊位投入使用，16—17号泊位、18—22号泊位、西湾航道建设加快推进。港口货物吞吐量达到5053万吨，增长49.4%，连续两年每年增加1000万吨以上，其中：防城港吞吐量达到3032万吨，增长21.0%，中小港口吞吐量达到2021万吨，增长1.3倍。集装箱完成17.31万标准箱，增长32.8％。港容港貌整治成效明显，港口综合管理水平进一步提高。

项目建设加快推进，投资拉动作用突出。全社会固定资产投资突破100亿元，达到103.53亿元，增长50.1%。沿海基础设施建设大会战进展顺利，沙潭江至企沙一级公路、企沙工业区路网等10个一期项目基本完成；防城南站至企沙铁路、江山至白龙二级公路等8个二期实施项目全部开工，完成总投资的64.0%。防城港企沙钢铁项目通过中咨公司的评估，前期工作扎实推进。防城港白龙核电项目的征地和“四通一平”工作进展顺利。投资对经济增长贡献率达到80％以上。

城镇建设明显加快，城市新格局初步形成。“还岛于港”取得新成效，城市基础设施进一步完善。干部群众期盼多年的行政中心正式启用。市中级法院、检察院、劳动力市场、国防教育基地等一批综合大楼基本竣工。临江新区规划等前期工作加快。房地产业快速发展，完成投资16.41亿元，增长2.03倍，增幅居全区首位。市污水处理厂、德城国际大酒店等一批项目开工建设。城市建成区达到32.09 平方公里，城镇化水平达到33.6%。深入开展城乡清洁工程，城乡面貌焕然一新。

新农村建设扎实推进，县域经济发展势头良好。预计农业总产值49.22亿元，增长6.3%。发展高效农业101.3万亩，完成造林8.74万亩。海水养殖、糖料蔗生产、肉桂、八角及优质水果等产业发展初具规模。农田水利、交通路网等农村基础设施不断完善。减轻农民负担6085万元，人均减负101.5元。县域经济实力明显增强，上思县被评为广西经济发展十佳县，防城区、东兴市荣获县域经济发展进步奖。

各项改革不断深化，对外开放取得新进展。农村综合改革逐步深化,财税、金融体制等改革稳步推进。非公有制经济加快发展。利用第四届中国—东盟博览会、2007中越边境（东兴—芒街）商贸·旅游博览会等经贸平台，引进区外境内资金24.45亿元，增长42.4%；直接利用外资5944万美元，增长19.7%。外贸进出口总额14.59亿美元，增长41.8%，边境贸易成交额75.12亿元，增长21.5%。与韩国永同郡正式结为友好城市。

经济社会协调发展，社会事业取得新进步。社会保障体系逐步完善，市、县两级城市低保标准提高，农村低保制度框架基本建立。教育“两基”工作顺利通过国家验收，贫困学生资助体系初步建立。城乡卫生服务体系建设加快，群众看病贵、看病难问题得到缓解。人口自然增长率控制在10.98‰以内。实施科技项目47项，引进推广农业新品种、新技术35项。农村文化致富工程、文化基础设施项目建设扎实推进。村村通广播电视无线覆盖一期工程竣工，新开通40个边远自然屯广播电视，解决20多万群众收听收看广播电视难问题。妥善处理历史遗留问题，偿还政府性债务2亿多元。各类安全生产事故同比下降12.31%。公安机关“三基工程”建设成效明显，刑事案件和治安案件持续下降。积极化解矛盾纠纷，群体上访事件明显减少。整顿市场秩序和打私工作有效开展。再次荣获全国双拥模范城称号。环境保护、审计、编制、物价、统计、外事、民族、宗教、档案、地方志、侨务、旅游、体育、新闻出版、气象、消防、防震减灾、机关后勤、残疾人事业、妇女儿童和老龄等各项工作取得新进展。

八件实事基本落实，民生问题进一步改善。认真办好市政府在市四届人大二次会议上郑重承诺的八件实事。华侨农林场的生产生活条件明显改善；“兴村富民”整村推进居广西前列，年内脱贫7166人；新型农村合作医疗参合人数达45万人；设立艾滋病防治基金；首期廉租住房项目开工建设，市金湾小区纳入临江新区建设统筹推进；新增城镇就业和转移农村剩余劳动力均超过1万人，城镇登记失业率为3.2%；社会保险覆盖范围扩大，基本养老保险等五项保险参保人数突破20万人；596名考上大学的贫困学生得到资助。

政府建设得到加强，服务能力明显提升。转变干部作风，加强机关行政效能建设取得阶段性成效，行政审批全面提速，投资环境进一步改善。市县两级政务服务中心建设顺利推进，东兴市荣获“全国政务公开工作先进单位”称号。反腐倡廉力度加大，纠风工作进一步强化。自觉接受人大和政协监督，办理市人大代表建议、批评和意见46件，办复率100%；办理市政协委员提案140件，办复率100%。

各位代表！

　　过去的一年，我市经济社会发展是在克服建设资金紧缺、天气持续干旱等诸多困难的情况下取得的，成绩来之不易！这是自治区党委、自治区人民政府及市委正确领导的结果，是市人大、市政协有效监督和大力支持的结果，是中直、区直单位和驻我市部队支持帮助的结果，是全市各族人民团结奋斗的结果。在此，我谨代表市人民政府，向全市广大干部群众，向解放军指战员、武警官兵和公安民警，向各民主党派、工商联、无党派人士、人民团体，向所有关心、支持、参与我市现代化建设的各界人士和海内外朋友，表示衷心的感谢和崇高的敬意！

回顾过去一年的工作，我们深深地体会到：只有认真贯彻落实科学发展观，抢抓新兴机遇，充分发挥比较优势，聚精会神搞建设，一心一意谋发展，才能加快发展、率先发展，最终实现富裕文明和谐防城港的奋斗目标。

在肯定成绩的同时，我们清醒地看到，发展中还面临不少困难和问题，突出表现为：经济总量小，经济要素分散，缺乏大型骨干企业带动，自主创新能力较弱；投融资体系尚不完善；城镇化发展滞后，中心城区规模小，集聚能力弱；农业现代化水平不高；人民生活水平较低；干部作风有待进一步转变，等等。对此，我们将高度重视，采取有效措施努力加以解决。

2008年总体工作安排

2008年，既是全面落实党的十七大精神的第一年，也是实施“十一五”规划承前启后的关键一年；既是奥运之年、我国改革开放30周年和自治区成立50周年，也是我市建市15周年和防城港建港40周年。做好今年的工作，意义重大，机遇难得。特别是国家批准实施《广西北部湾经济区发展规划》，标志着广西北部湾经济区的开放开发正式纳入国家发展战略，标志着广西北部湾经济区进入新的发展阶段，标志着广西将掀起建设中国沿海地区经济新高地和发展新一极的高潮，广西北部湾经济区将建设成为重要国际区域经济合作区，历史将掀开崭新篇章！

根据中央、全区、全市经济工作会议精神和市委的总体部署，今年政府工作的总体要求是：全面贯彻落实党的十七大精神，高举中国特色社会主义伟大旗帜，以邓小平理论和“三个代表”重要思想为指导，以科学发展观为统领，围绕“三个率先”目标，坚持以港兴市、以工业兴市、以区位优势兴市，全面推动广西北部湾经济区发展规划的实施，大力推进工业化、城镇化，加快基础设施和重大项目建设，着力改善民生，促进社会和谐，推动经济社会又好又快发展，为早日把我市建成广西重要的钢铁工业基地、商贸物流基地、能源基地、进出口加工制造基地、国际旅游胜地和连接多区域的国际通道、交流桥梁、合作平台，为加快建设开放度高、辐射力强、经济繁荣、社会和谐、生态良好的防城港而努力奋斗。

全市经济社会发展的主要目标是：生产总值增长20%以上；财政收入增长25%以上；全社会固定资产投资增长30%以上；工业总产值增长30%以上；全市港口货物吞吐量增长20%以上；社会消费品零售总额增长16%以上；外贸进出口总额增长25%以上；外商直接投资增长20%以上；区外境内招商引资到位资金增长25%以上；城镇居民人均可支配收入增长12%以上；农民人均纯收入增长10%以上；城镇新增就业人数9500 人，城镇登记失业率控制在4%以内；人口自然增长率控制在11.2‰以内；万元生产总值能耗、主要污染物排放量控制在自治区要求范围内。

当前，虽然我们面临发展的重大历史机遇，但也要看到建设用地偏紧、建设资金紧缺、节能减排压力增大等形势，看到我市经济社会发展存在的困难和问题，始终保持清醒头脑，增强忧患意识，全面落实科学发展观，坚持“好字优先、好中求快、稳中求进、紧中求活”，进一步解放思想，创新发展理念，转变发展方式，努力实现速度与质量、效益相统一，促进经济社会又好又快发展。

在总量扩张中不断提升经济发展质量

2008年，要紧紧抓住广西北部湾经济区的开放开发纳入国家发展战略的重大历史机遇，认真开展新一轮思想大解放活动，明确定位，发挥优势，在全市迅速掀起新一轮大发展的热潮，争当广西北部湾经济区开放开发的排头兵。为此，要重点抓好以下几项工作：

一、率先推进，全面实施广西北部湾经济区发展规划

解放思想抢抓机遇。以国家批准实施《广西北部湾经济区发展规划》为契机，深入开展新一轮思想大解放讨论活动，解放思想，开拓创新，敢为人先，敢想敢干。以只争朝夕的精神抢抓机遇，以超常思维、更宽视野、更高层面谋划发展；最大限度地调动一切积极因素，激发全民创业激情，齐心协力、万众一心促进发展。

深入领会规划内容。深入研究、解读、领会《广西北部湾经济区发展规划》的内容，牢牢把握规划对我市产业发展、城镇建设、对外开放等诸多领域的新要求，找准功能定位，充分发挥优势，扎实推进我市钢铁工业、商贸物流、进出口加工、能源、旅游等方面工作，真正建成连接多区域的国际通道、交流桥梁、合作平台。

深化细化专项规划。抓紧研究制订实施《广西北部湾经济区发展规划》的工作方案和行动计划，抓紧修编城市总体规划、土地利用总体规划、海洋功能区划和港口布局规划，完善东湾工业区、企沙工业区等规划，开展大型综合物流基地等规划编制，以扎实细致的工作做好规划对接、产业对接、项目对接和政策对接。

推进钢铁、核电项目取得实质性进展。防城港钢铁项目、白龙核电项目是我市全面推进《广西北部湾经济区发展规划》实施的“天字号工程”，是事关我市经济社会全局，实现跨越发展举足轻重的两大核心项目。按照三年出产品，三年见效益的目标，排出工作时间表，积极配合项目业主，全面启动钢铁项目建设。同时，积极推进白龙核电项目基础工程建设步伐，全力做好项目前期工作。

二、深入实施项目带动战略，保持经济强劲增势

今年是我市的“项目建设推进年”，要按照市委的部署，坚持以项目建设为中心，大力推进临海临港工业建设，完善基础设施，保持投资对经济增长的强劲拉动。

突出抓好项目建设。要坚持大中小项目并举、基础设施与产业项目并举、高新项目与传统项目并举、内资项目与外资项目并举、沿海项目与内地项目并举，以只争朝夕的精神，领导挂帅、全民动员，狠抓项目工作。围绕重大产业布局，加快能源、钢铁、化工、粮油食品加工和物流等临海临港重大产业项目建设。全面建成防城南站至企沙铁路、江山至白龙二级公路等沿海基础设施建设大会战二期项目。加快市污水处理厂等民生工程建设，确保在自治区成立50周年大庆前全部竣工。加强项目前期工作，协调解决项目征地用地、报批审批等重大问题，超前做好项目储备。

壮大新型工业经济。继续发挥港口优势，充分利用两个市场、两种资源，大力改善发展环境，突出抓好大海粮油扩建工程、益海大豆、农垦防城分公司精制糖等项目建设，推进中电防城港电厂二期等项目前期工作。抓好企业技术改造，提高自主创新能力。加强煤电油运的综合调度。市财政投入2000万元作为资本金，搭建融资平台，重点加快企沙工业区和东兴江平、港口公车、防城茅岭、上思城西等工业集中区的基础设施建设，建立健全园区管理体制和综合服务机制，努力培育临海临边优势产业集群。

持续增强港口核心竞争力。重点建设防城港18—22号泊位、东湾403—407号泊位、3个企沙钢铁码头和东湾、西湾航道工程。开拓一批内外贸航线，改善集疏运条件。做好岸线资源特别是企沙半岛沿岸的规划和开发，加快白龙港、企沙港等一批中小港口建设，形成大中小港口齐头并进的良好局面。

三、着力增强城市功能，加快城镇化进程

依托港口开发和临港工业促进城市发展，到2020年，城市建成区人口发展到50～60万人，建设用地控制在70平方公里内。为此，要继续实施“还岛于港”战略，加快推进城市中心区建设，北联南接，向东拓展，充分利用“三岛三湾”美丽的自然风光和优良的生态环境，把我市建设成为适宜居住、利于创业的大城市；加快重点镇建设，形成特色鲜明的城镇体系。

高标准做好城市规划和管理。以城市中心区规划为重点，统筹规划港口区、防城区和东兴市，整合资源，优化要素配置，精心构建大城市发展框架。特别要做好西湾岸线等城市重要地段、重要节点的规划设计，抓紧实施城市亮化、绿化、美化工程，完善城市景观，提高城市品位。加强城镇综合管理，深入开展“城乡清洁工程”。

加快推进以城市中心区为重点的城市建设。争取全年市政基础设施建设投入突破20亿元，着力加强供水、供电、供气、道路、照明、排水排污等城市基础设施建设，推进东西大道、滨海大道等道路建设，进一步完善行政中心区配套设施，加快推进临江新区开发。市中级法院、检察院、劳动力市场、国防教育基地等一批项目要在年内建成投入使用。加紧打造新区商业中心，加快星级宾馆建设，改善旅游接待环境。逐步配套完善西湾等居民区的公共服务功能。加快“城中村”改造。

四、发展壮大县域经济，扎实推进“三农”工作

创新工作思路，加大资金倾斜，扎实推进社会主义新农村建设，为我市在全区率先解决“三农”问题，率先实现全面小康社会目标奠定基础。

加快县域经济发展。以争创全区县域经济发展十佳县为契机，积极推进县域工业化、城镇化、农业产业化，大力发展特色产业和农业龙头企业，努力形成“一乡一业，一村一品”，全面增强县域经济的活力和竞争力。

大力发展现代农业。在稳定粮食种植面积的基础上，重点支持临海地区发展海水生态养殖、远洋捕捞和海产品加工业；支持城乡结合部地区发展商贸和蔬菜种植等产业；支持十万大山腹地农村发展甘蔗、八角、肉桂、木薯等优势产业。充分发挥农业的多种功能，发展特色高效农业，拓宽农民致富路子。加强重大疫情防控。实施“菜篮子”工程，让市民买到放心菜、平价菜。

加强农业基础设施建设。加强农村路网、电网、信息网等建设，建立健全农业社会化服务体系。抓好病险水库除险加固等水利设施建设，逐步完善防汛防台风和抗旱减灾体系。

加大支农惠农力度。进一步落实扶持生猪生产、水稻良种补贴等各项支农惠农政策。加强对农村合作经济组织的扶持。克服冷冻灾害影响，抓好灾后恢复生产。深入开展驻村帮扶工作，扎实推进新农村建设。

五、进一步深化改革，不断提高开放合作水平

深化各项改革。继续推进国有企业等重点领域和关键环节改革，逐步健全国有资产监督管理体制。深化资本、土地、劳动力市场等要素市场体制改革和文化体制、医疗卫生体制、社会体制等改革。创造条件积极推进农村综合改革，抓好华侨农林场改革与发展。加快农村信用社改革，推进各类投融资平台建设。建立健全社会信用体系，整顿和规范市场经济秩序，营造公平竞争的市场环境。

推动全民创业。完善支持引导非公经济发展的体制机制，推进公平准入，清理和修订不利于非公有制经济发展的政策性文件，确保非公有制企业在投融资、财税、信贷等方面，与其他企业享受同等的待遇，以创业促就业、促发展。

开创招商引资新局面。抓住广西北部湾经济区的开放开发上升为国家发展战略的机遇，认真研究和利用好国家宏观调控政策，在创新招商引资机制、拓宽投融资渠道和招大商、引大商方面下大功夫，努力把我市建设成为吸引外来投资者和承接产业转移的重要基地。采取产业招商、以商招商、小分队招商等方式，重点加大与国有大型企业及江浙、粤港澳等东部发达地区的合作力度。坚持公开、透明、平等的优惠政策。健全招商项目落户机制，切实提高项目履约率。全年引进资金要突破30亿元。

提高对外开放水平。充分利用中国—东盟博览会、中越边境（东兴—芒街）商贸·旅游博览会和友好城市等平台，扩大区域合作，重点加强与东盟国家贸易往来和港口物流等方面的合作。

六、加强财税工作，增强资金保障能力

狠抓财政增收。抓好重大项目、重点企业、重点行业的税源培育，做大做强支柱财源。做好土地营销文章，为发展提供资金保障。认真研究财政政策，积极争取上级支持，努力拓展外生财源。强化收入征管，依法治税，依率计征，应收尽收。加强非税收入管理，严格执行收支两条线。建立健全收入增长激励机制，促进各级财政又好又快增长。

加强支出管理。深化部门预算、政府采购、国库集中支付制度等改革。建立科学、规范、高效的财政资金分配和管理体系，切实提高资金使用效率。优化支出结构，确保各项重点支出，特别要加大对“三农”工作、市政公共设施、社会保障、节能减排等领域和教育、卫生等公共事业的投入，抓好规范公务员津贴补贴工作。

七、大力发展服务业，积极扩大消费需求

打造商贸物流基地。按照构建环北部湾区域性物流中心的要求，以大港口为依托、以大工业为支撑、以生产性物流为方向，整合物流资源，优化物流管理，完善物流体系，积极培育沿海沿边专业市场，发展壮大现代物流业。大力发展边境贸易、边境出口加工，建设边境商贸物流基地。深入实施电子口岸和“大通关”工程，逐步规范物流市场，实现物畅其流。

发展新兴服务业。积极构建北部湾旅游圈，打造“山海边”和民俗旅游品牌，高标准规划，高起点实施整体开发，推进一批景区景点建设，争创中国优秀旅游城市，培育成为区域性国际旅游胜地。引导开发商有序盘活土地，开发高品质住宅，促进房地产业健康发展。加强融资平台建设，促进政银企合作，改善金融生态环境，提高金融服务水平，积极发展法律、会计、评估、社区服务等服务业，培育新的经济增长点。

扩大消费需求。持续增加城乡居民收入，建立健全职工工资正常增长和支付保障机制，确保农民工工资按时足额发放。改善城乡消费环境，加快商贸流通体系建设。积极发展旅游、文化、体育等服务性消费，鼓励和引导居民扩大住房、汽车、教育、保健等消费，推动消费结构升级。

全面推进社会和谐发展

按照党的十七大报告提出的“学有所教，劳有所得，病有所医，老有所养，住有所居”的要求，坚持以人为本，高度关注民生，建立健全改善民生的长效机制，积极营造共建共享和谐的生动局面。

一、着力解决民生问题

积极促进社会就业。不断拓宽就业渠道，通过大企业、新项目的落户，用发展的方式来解决就业问题。强化公共就业服务，大力开展创建“充分就业社区”活动，巩固消除“城镇零就业家庭”成果。多渠道、多形式地培养一批有文化、有专业技能的新型农民，年内新增农村劳动力转移就业1万人。

完善社会保障体系。积极推进全市基本养老、医疗、失业、工伤和生育保险全覆盖，构筑全方位的社会保障安全网。启动实施城镇居民参加基本医疗保险工作。探索建立农村养老保险制度，加强“五保村”建设，做好“五保户”集中供养。抓好社会福利、社会救济、慈善互助和优抚工作。设立扶助基金，建立贫困学生多元扶助制度。关心弱势群体和特困群众的生活，维护征地拆迁失地群众的合法权益，初步建立被征地群众社会保障机制，妥善处理拆迁安置问题。维护市场价格秩序，落实物价上涨补助措施，保障低收入群众基本生活。

二、努力建设生态文明

坚持节约资源和保护环境的基本国策，深入开展节能减排全民行动，提高全社会节能减排意识，大力建设资源节约型、环境友好型社会。市财政投入2000万元，用于节能减排和环境保护。强化节能减排目标责任制，尽快建立实施节能减排统计、监测和考核评价体系，实行严格的问责制和一票否决制。建立健全节能减排激励机制、约束机制和淘汰落后产能退出保障及补偿机制。抓好火电、制糖、钢铁、建筑、交通等领域的节能减排和技术改造，开展污染源普查，全面推行清洁生产。重点加强防城江、明江流域环境综合治理，切实保护好十万大山和东湾、西湾、珍珠湾生态环境。加强计划生育依法行政工作，进一步稳定低生育水平。加大存量用地盘活力度，妥善解决历史遗留问题；推进土地利用总体规划和海洋功能区划修编；按照节约集约的要求，统筹各类建设用地。

三、全面发展社会事业

优先发展教育科技。优化教育资源配置，确保教育经费足额投入，推动城乡教育协调发展。全面实施素质教育。集中力量开展职业教育攻坚战。积极实施科技创新项目，提高科技对经济增长的贡献率。深入实施人才强市战略，尊重知识、尊重人才，打造群贤毕至、才智涌动、活力迸发的人才高地。

加快发展卫生事业。积极推进市第一人民医院“创三甲”工作。完善农村三级卫生服务网络和城市社区卫生服务体系。切实做好重大传染病防控。加强食品、药品安全监管。纠正行业不正之风，减轻群众就医负担。

大力发展文化生产力。设立宣传文化专项资金，完善公共文化服务体系，积极推进县、乡、村三级文化设施和市文化活动中心、公共图书馆建设，建成东兴京族生态博物馆。继续实施农村文化致富工程，全面推进基层文化建设。深入挖掘民族和地方文化资源精品，加强文物管理和非物质文化遗产保护。进一步规范文化市场秩序。广泛开展全民健身迎奥运活动，丰富群众文体生活。扩大以东盟国家为重点的对外文化交流，加强对外宣传，树立良好的城市形象。

发展其他各项事业。关心支持残疾人事业，重视做好妇女儿童和老龄工作。加强审计、统计、编制、气象、消防、外事、侨务、民族、宗教、档案、地方志、新闻出版、防震减灾、机关后勤等各项工作，推进社会事业全面进步。

四、提高社会管理水平

维护社会稳定安全。强化社会治安综合治理，深入开展严打整治斗争，努力建设“平安防城港”。建立健全各种突发事件应急处理机制，提高保障公共安全和处理突发事件能力。建立长效机制，抓好打击走私、贩毒和跨境赌博等工作。积极预防和化解人民内部矛盾，妥善处理群体性事件。严格落实安全生产责任制，抓好重点行业和领域的专项整治，预防重特大事故发生。

做好双拥工作。加强国防动员、人民防空、交通战备、边海防和民兵预备役建设，深入开展拥军优属、军民共建、爱民固边活动，巩固和发展军政军民团结。

抓好精神文明建设。加强社会公德、职业道德、家庭美德教育和未成年人思想道德建设，倡导科学、文明、健康的生活方式。加强网络文化建设和管理。推进边海文明示范带、文明社区等群众性文明创建工作。

五、办好事关民生的八件实事

各位代表！为民办实事是人民政府义不容辞的责任。今年要进一步建立健全为民办实事的长效机制，以解决人民群众最关心、最直接、最现实的利益问题为重点，集中力量办好八件实事：

1．实施改善农村生产生活环境工程。市财政安排2200万元，重点抓好农村道路、饮水安全、沼气池、供电、广播电视、垃圾污水处理、绿化美化和文化、体育场所建设。

2．实施兴边富民工程。重点解决离边境线3公里范围内边境地区村屯的交通、饮水、用电、就医、上学、住房等困难问题，加强边境一线村屯的基础设施建设，改善群众生产生活条件。

3．实施城乡最低生活保障工程。逐步提高城乡最低生活保障标准，城市低保人员月人均补差提高到115元，农村低保人员月人均补差不低于25元，将符合条件的贫困人口全部纳入保障范围。

4．实施教育惠民工程。市财政安排800万元，改造和建设食堂、厕所、卫生室、运动场等一批校园基础设施；市财政安排800万元，建设市职业教育中心。

5．实施医疗惠民工程。进一步推行和完善新型农村合作医疗制度，实现四个区、县（市）全覆盖，参合率提高到85%；从今年起，市财政每年安排一定的专项经费，建立健全大病救助制度，对城乡没有收入来源、患重大疾病的居民，给予治疗补助；市财政安排600万元，抓好乡镇中心卫生院和村级医疗点建设，逐步改善农村医疗卫生条件。

6．实施困难归侨和农村特困户住房改造工程。多方筹措资金，解决170户困难归侨的危旧房改造；逐步解决农村特困户和残疾人危房改造问题。

7．实施廉租房保障工程。积极筹措资金，建成廉租住房面积6000平方米，逐步解决城市低收入家庭住房难问题。

8．实施安居工程。年内动工建设金湾小区，分期分批解决市直干部职工的住房难问题。

努力建设服务型政府

按照党的十七大提出建设服务型政府的新要求，结合开展行政效能建设“回头看”和思想大解放活动，进一步加强政府自身建设，继续推进行政管理体制改革和政府职能转变，全面提升服务水平。

求真务实，为民行政。大力弘扬求真务实之风，坚决反对形式主义、官僚主义，着力精简会议、精简文件，深入基层调查研究，倾听群众呼声，反映群众愿望，关心群众疾苦，多为群众办好事、办实事，做到权为民所用、情为民所系、利为民所谋。

创新管理，高效行政。着力转变职能、理顺关系、优化结构、提高效能，形成权责一致、分工合理、务实高效、执行顺畅、监督有力的行政管理体制。完善市、县两级政务服务中心。加快电子政务建设。落实责任制、问责制和约束激励机制，严格执行政府工作规则，严肃政府工作纪律，提高政府的公信力和执行力。

健全机制，依法行政。加快法治政府建设进程，建立健全决策、执行、监督相协调的政务机制。自觉接受人大及其常委会的法律监督和工作监督，自觉接受政协的民主监督以及社会舆论监督。充分发挥工会、共青团、妇联等群团组织的桥梁纽带作用。巩固和扩大基层民主。加强政务公开，推进决策科学化、民主化。

规范运作，廉洁行政。严格执行党风廉政建设责任制，深入开展反腐倡廉工作。规范权力运行，加强对土地出让、建设工程招投标等重点环节的管理和监督，营造风清气正的施政环境。

各位代表！

新的一年，孕育着新的希望。防城港市正站在新的起跑线上，形势催人奋进，任务艰巨而光荣。让我们在市委的正确领导下，紧紧抓住实施《广西北部湾经济区发展规划》的重大历史机遇，团结带领全市各族人民，坚定信心，振奋精神，解放思想，开拓创新，以优异的成绩向我国改革开放30周年和自治区成立50周年献礼!
